# Association of the Charlson Comorbidity Index and Age-Adjusted Charlson Comorbidity Index with Clinical Course and Mortality in Patients with Idiopathic Pulmonary Fibrosis

**DOI:** 10.3390/biomedicines14102176

**Published:** 2026-09-25

**Authors:** Tugba Onyilmaz, Gozde Oksuzler Kizilbay, Huseyin Kaya, Serap Argun Baris, Bengugul Ozturk, Hasim Boyaci, Ilknur Basyigit

**Affiliations:** 1 Department of Pulmonary Medicine, Faculty of Medicine, Kocaeli University, 41380 Kocaeli, Turkey; asli_tugba@hotmail.com (T.O.); dr.hsynkaya@hotmail.com (H.K.); serapargun2002@yahoo.com (S.A.B.); rrockunzell@gmail.com (B.O.); haboyaci@yahoo.com (H.B.); ilknur.basyigit@gmail.com (I.B.); 2 Department of Pulmonary Medicine, Private Konak Hospital, 41100 Kocaeli, Turkey

**Keywords:** idiopathic pulmonary fibrosis, Charlson Comorbidity Index, age-adjusted Charlson Comorbidity Index, comorbidity, clinical outcomes

## Abstract

**Background/Objectives:** Idiopathic pulmonary fibrosis (IPF) frequently coexists with comorbidities that may influence clinical outcomes. The Charlson Comorbidity Index (CCI) quantifies the weighted comorbidity burden, whereas the age-adjusted Charlson Comorbidity Index (ACCI) additionally incorporates age. We evaluated the associations of CCI and ACCI with pulmonary function, disease severity, clinical course, and mortality in IPF. **Methods:** This retrospective, single-center cohort study included 319 patients with IPF. Comorbidities present at diagnosis were scored using the CCI and ACCI. Associations with pulmonary function, GAP index, acute exacerbations, hospitalization, long-term oxygen therapy, echocardiographic findings suggestive of pulmonary hypertension, incident malignancy, antifibrotic treatment, and mortality were assessed. Correlation analyses were performed using Spearman’s rank correlation coefficient, and Bonferroni correction was applied to account for multiple testing. Multivariable Cox proportional hazards regression was used to evaluate whether the CCI or ACCI was independently associated with survival. Time-to-event analyses were performed using Kaplan–Meier survival analysis. **Results:** The mean age was 71 ± 8 years, 78.1% were male, and 75.5% had at least one comorbid condition among patients with available comorbidity data. The median CCI and ACCI scores were 2 (IQR, 0–4) and 5 (IQR, 4–7), respectively. Higher CCI and ACCI categories were associated with hospitalization (*p* = 0.006 and *p* = 0.011), echocardiographic findings suggestive of pulmonary hypertension (both *p* = 0.002), and incident malignancy (*p* < 0.001 and *p* = 0.005, respectively). After Bonferroni correction, higher CCI and ACCI categories remained significantly associated with echocardiographic findings suggestive of pulmonary hypertension (both *p* = 0.002) and incident malignancy (both *p* < 0.001), whereas the associations with hospitalization did not remain statistically significant. A nominal association between ACCI and mortality (*p* = 0.044) also did not remain significant after Bonferroni correction. In Spearman analyses, ACCI remained significantly correlated with current age, age at IPF diagnosis, and the GAP index after correction, whereas the correlations of CCI and ACCI with baseline DLCO did not. Neither CCI (HR = 1.029, 95% CI: 0.923–1.146; *p* = 0.610) nor ACCI (HR = 1.035, 95% CI: 0.936–1.144; *p* = 0.509) was independently associated with survival in multivariable Cox regression analyses. Lower baseline DLCO remained independently associated with survival in both Cox models (both *p* < 0.001).Kaplan–Meier survival curves were constructed according to the predefined CCI and ACCI score categories (0, 1–2, 3–4, and ≥5), and survival distributions across categories were compared using the log-rank test. **Conclusions:** CCI and ACCI were associated with selected clinical characteristics in IPF, particularly echocardiographic findings suggestive of pulmonary hypertension and incident malignancy. However, neither index independently predicted survival after multivariable adjustment. These indices may complement, rather than replace, disease-specific physiological assessments in patients with IPF.

## 1. Introduction

Idiopathic pulmonary fibrosis (IPF) is a chronic, progressive, fibrosing interstitial lung disease of unknown cause that occurs predominantly in older adults and is associated with progressive deterioration in lung function, worsening respiratory symptoms, and high mortality [1,2].Disease progression varies considerably among individuals with IPF, with some patients experiencing gradual functional deterioration and others showing rapid progression or acute episodes of respiratory decline. Pirfenidone and nintedanib have improved the pharmacological management of IPF by reducing the rate of decline in forced vital capacity (FVC); however, neither treatment is curative, and progressive disease remains an important clinical challenge [2,3,4].

In addition to the underlying fibrotic lung disease, patients with IPF frequently have multiple respiratory and non-respiratory comorbidities. Cardiovascular diseases, pulmonary hypertension, emphysema, other chronic respiratory disorders, gastroesophageal reflux disease, diabetes mellitus, obstructive sleep apnea, and malignancies are among the conditions commonly encountered in this population [5,6]. The high prevalence of multimorbidity is particularly relevant because IPF predominantly affects older individuals, in whom the burden of chronic diseases generally increases with age. Comorbid conditions may contribute to symptom burden, impair quality of life, complicate clinical management, and influence long-term outcomes [5,6]. Consequently, the assessment of IPF based solely on the severity of pulmonary involvement may not fully reflect the overall clinical burden of the disease.

Several approaches have been proposed to improve the prognostic assessment of IPF. The Gender–Age–Physiology (GAP) index incorporates sex, age, FVC, and diffusing capacity of the lung for carbon monoxide (DLCO) to provide a disease-specific estimate of the mortality risk [7]. However, these physiological models do not directly quantify the overall burden of coexisting diseases. The Charlson Comorbidity Index (CCI) was originally developed as a weighted method for classifying comorbid conditions according to their association with mortality and has subsequently become one of the most widely used measures of comorbidity burden [8]. Because age independently contributes to mortality risk, the original index was later combined with an age component, resulting in the age-adjusted Charlson Comorbidity Index (ACCI) [9]. This distinction may be particularly relevant in IPF, given the strong association between the disease and advanced age.

Comorbidity-based indices have been increasingly applied in IPF and other interstitial lung diseases to complement conventional clinical and physiological measures [10,11,12]. Although CCI and comorbidity burden have previously been evaluated in IPF and other ILDs, direct comparison of the conventional CCI and ACCI within the same real-world IPF cohort across multiple clinical and functional outcomes remains clinically relevant. Accordingly, the present study aimed to evaluate the associations of CCI and ACCI with demographic and functional characteristics, disease severity, clinical course, including acute exacerbations, hospitalization, long-term oxygen therapy requirement, and mortality in patients with IPF.

## 2. Materials and Methods

### 2.1. Study Design and Population

This single-center, retrospective cohort study was conducted at the Department of Pulmonology, Kocaeli University Faculty of Medicine Hospital. The medical records of patients diagnosed with IPF between January 2015 and November 2025 were retrospectively reviewed for this study. Potentially eligible patients were identified through the hospital information system using the International Classification of Diseases, Tenth Revision code J84.1, and the diagnosis of IPF was verified by reviewing their medical records. All eligible patients within the study period were included; therefore, no formal a priori sample size calculation was performed.

Patients were eligible if they were older than 18 years and had received a diagnosis of IPF following evaluation by a multidisciplinary interstitial lung disease team. Diagnosis was established based on clinical, radiological, and, when available, histopathological findings in accordance with international diagnostic recommendations [1,2]. Eligible patients had a definite or probable usual interstitial pneumonia (UIP) pattern on high-resolution computed tomography and/or histopathological findings compatible with UIP after excluding other known causes of interstitial lung disease (ILD).

Patients with a UIP pattern attributable to a condition other than IPF, including asbestosis, hypersensitivity pneumonitis, connective tissue disease-associated interstitial lung disease, drug-induced lung injury, or radiation-induced lung injury, were excluded.

### 2.2. Data Collection and Clinical Outcomes

Demographic and clinical data were obtained from electronic and archived medical records using a standardized data collection form. The recorded variables included sex, age, age at IPF diagnosis, duration of follow-up, pharmacological treatment, use of anti-fibrotic therapy, and the prescribed anti-fibrotic agent (pirfenidone or nintedanib).

The pulmonary function test parameters included forced expiratory volume in one second (FEV_1_), FVC, FEV_1_/FVC ratio, and DLCO. Measurements obtained at baseline, approximately 6 and 12 months after diagnosis, and at the most recent follow-up were recorded.

The Gender–Age–Physiology (GAP) index was calculated using sex, age, baseline FVC percentage predicted, and baseline DLCO percentage predicted, as originally described by Ley et al. [7]. The resulting GAP scores were classified as stage I (0–3 points), stage II (4–5 points), or stage III (6–8 points). The GAP index was calculated only for patients for whom all required baseline variables were available.

The clinical course was evaluated according to the occurrence and annual frequency of acute exacerbations, requirement for hospitalization, number and duration of hospitalizations, requirement for long-term oxygen therapy, pulmonary hypertension, development of a new malignancy after IPF diagnosis, and mortality. Pulmonary hypertension was assessed using transthoracic echocardiography. Patients with an estimated systolic pulmonary artery pressure (sPAP) ≥40 mmHg on transthoracic echocardiography were classified as having echocardiographic findings suggestive of pulmonary hypertension for the purposes of the present study [13]. Right heart catheterization data were available only for a subset of patients and were therefore not required for classification. Follow-up duration and survival time were calculated from the date of IPF diagnosis to death or the last clinical assessment date. Clinical events and outcomes were identified according to the diagnoses and events documented in the medical records.

### 2.3. Charlson Comorbidity Index and Age-Adjusted Charlson Comorbidity Index

Comorbidities present at the time of IPF diagnosis were identified from the patients’ medical records and scored using the original Charlson Comorbidity Index. The CCI assigns predefined weights of 1, 2, 3, or 6 to 19 comorbidities according to their association with mortality. The individual weights were summed to obtain the total CCI score, which represents the overall comorbidity burden, independent of age [8].

The Age-Adjusted Charlson Comorbidity Index was calculated by adding an age-related score to the conventional CCI based on the patient’s age at the time of IPF diagnosis. In accordance with the classical age-adjustment method, no additional points were assigned to patients aged ≤40 years, whereas 1 point was added for those aged 41–50 years, 2 points for those aged 51–60 years, 3 points for those aged 61–70 years, and 4 points for those aged ≥71 years [9]. Accordingly, the maximum possible scores were 33 and 37 for the CCI and the ACCI, respectively.

For categorical analyses, both the CCI and ACCI scores were classified as follows: 0 points, no comorbidity; 1–2 points, mild comorbidity; 3–4 points, moderate comorbidity; and ≥5 points, severe comorbidity. The indices were also evaluated as continuous scores in the correlation analysis.

### 2.4. Statistical Analysis

Statistical analyses were performed using IBM SPSS Statistics, version 25.0 (IBM Corp., Armonk, NY, USA). The distribution of continuous variables was assessed using the Kolmogorov–Smirnov test. Normally distributed continuous variables were presented as mean ± standard deviation, whereas non-normally distributed variables were summarized as median and interquartile range. Categorical variables were reported as frequencies and percentages.

For comparisons between two independent groups, the independent-samples Student’s *t*-test was used for normally distributed variables, and the Mann–Whitney *U* test for non-normally distributed variables. Comparisons involving more than two groups were performed using one-way analysis of variance or the Kruskal–Wallistest, as appropriate. Correlations between CCI or ACCI scores and continuous demographic, clinical, and pulmonary function variables were assessed using Spearman’s rank correlation coefficient (ρ), considering the ordinal and non-normally distributed nature of the comorbidity scores and several clinical variables. Time-to-event analyses were additionally performed using Kaplan–Meier survival analysis. Kaplan–Meier survival curves were constructed according to the predefined CCI and ACCI score categories (0, 1–2, 3–4, and ≥5), and survival distributions across categories were compared using the log-rank test. Multivariable Cox proportional hazards regression analyses were performed to evaluate whether CCI or ACCI was independently associated with survival. Survival time was defined as the interval from IPF diagnosis to death or the last available clinical assessment. Two separate models were constructed. In the CCI model, CCI was adjusted for age at diagnosis, baseline FVC % predicted, baseline DLCO % predicted, and antifibrotic treatment. Because age is intrinsically incorporated into the ACCI, age was not entered as a separate covariate in the ACCI model to avoid redundancy and potential collinearity; therefore, the ACCI model was adjusted for baseline FVC % predicted, baseline DLCO % predicted, and antifibrotic treatment. Hazard ratios (HRs) with 95%confidence intervals (CIs) were reported. Cox regression analyses were performed using complete cases for the variables included in each model. Analyses were conducted using the available observations for each variable, resulting in variable-specific denominators. Given the substantial missing data for baseline DLCO and the GAP index, a missing-data assessment was performed. The proportions of available and missing observations were summarized for the principal pulmonary function and disease severity variables. Patients with and without available baseline DLCO and GAP data were compared with respect to demographic characteristics, comorbidity indices, treatment, and clinical outcomes. In addition, to evaluate the robustness of the survival findings for missing baseline DLCO data, sensitivity Cox proportional hazards regression analyses were performed after excluding baseline DLCO from the primary models. All statistical tests were two-sided in nature. To account for multiple tests, the Bonferroni correction was applied within prespecified families of related comparisons. For the clinical comparisons performed separately for the CCI and ACCI, 10 comparisons were evaluated for each index, resulting in a Bonferroni-corrected significance threshold of *p* < 0.005 (0.05/10). For the correlation analyses, 14 correlations were evaluated separately for each index, resulting in a Bonferroni-corrected significance threshold of *p* < 0.0036 (0.05/14). Unadjusted *p*-values are reported, and statistical significance was interpreted according to the corresponding Bonferroni-corrected thresholds.

## 3. Results

### 3.1. Patient Characteristics and Clinical Outcomes

A total of 319 patients with idiopathic pulmonary fibrosis were included in this study. Of these, 249 (78.1%) were men and 70 (21.9%) were women. The mean age of the study population was 71 ± 8 years, and the mean age at IPF diagnosis was 66 ± 5 years old. Overall, 120 patients (37.6%) were diagnosed before the age of 65 years, whereas 199 (62.4%) were diagnosed at ≥65 years of age. The mean GAP index was 3.6 ± 1.4 (Table 1).

At least one comorbid condition was identified in 241 patients (75.5%). Considering all recorded comorbidities, the most frequently observed conditions were hypertension (64.1%), coronary artery disease (43.4%), and chronic obstructive pulmonary disease (30.3%). The distributions of the remaining comorbidities are presented in Table 2 and Figure 1. The median Charlson Comorbidity Index (CCI) score was 2 (interquartile range [IQR], 0–4). Based on the CCI categories, 86 patients (27.0%) had no comorbidity burden, 99 (31.0%) had a mild burden, 76 (23.8%) had a moderate burden, and 58 (18.2%) had a severe burden of comorbidities. The median Age-Adjusted Charlson Comorbidity Index (ACCI) score was 5 (IQR, 4–7). According to the ACCI classification, 17 patients (5.3%) had a mild burden, 116 (36.4%) had a moderate burden, and 185 (58.0%) had severe burden.

Baseline pulmonary function tests showed a mean FEV_1_ of 2175 ± 647 mL (80 ± 18% predicted), mean FVC of 2639 ± 824 mL (76 ± 18% predicted), and mean FEV_1_/FVC ratio of 84 ± 11%. The mean DLCO was 51 ± 16% predicted (Table 1).

Antifibrotic treatment was administered to 221 patients (70.2%). Of the treated patients, 125 (56.6%) received pirfenidone and 96 (43.4%) received nintedanib treatment. During follow-up, 51 patients (16.0%) experienced at least one acute exacerbation, and 128 (40.1%) required hospitalization. The mean length of hospital stay was 14 ± 20 days. Long-term oxygen therapy was prescribed to 120 patients (37.7%). The mean time elapsed since IPF diagnosis was 43.8 ± 26.6 months, and 124 patients (38.9%) died during the study period.

### 3.2. Associations of CCI and ACCI with Clinical Characteristics and Outcomes

The distributions of the CCI and ACCI categories did not differ significantly according to sex. Similarly, neither index differed according to the receipt of antifibrotic treatment or the type of antifibrotic agent administered. No significant differences in CCI or ACCI were observed according to the history of acute exacerbations. In contrast, both indices differed significantly according to age at IPF diagnosis (both *p* < 0.001).

Patients who required hospitalization showed higher CCI and ACCI scores than those who did not (*p* = 0.006 and *p* = 0.011, respectively); however, these associations did not remain statistically significant after Bonferroni correction (corrected significance threshold, *p* < 0.005). The hospitalization rate increased to 51.7% in patients with severe CCI. Furthermore, 66.4% of all hospitalized patients had a severe comorbidity burden according to the ACCI.

Both indices were significantly associated with incident malignancies during the follow-up period, and these associations remained significant after Bonferroni correction. Among patients who developed a new malignancy, 74.2% and 87.1% were classified as having severe CCI and ACCI scores, respectively (*p* < 0.001 for both).

CCI and ACCI scores were also significantly higher in patients with echocardiographic findings suggestive of pulmonary hypertension (*p* = 0.002 for both), and these associations remained statistically significant after Bonferroni correction. No significant differences in either index were identified according to the requirement for long-term oxygen therapy.

A nominal association was observed between ACCI and mortality (unadjusted *p* = 0.044); however, this association did not remain statistically significant after Bonferroni correction. Of the patients who died, 62.9% were classified as having a severe ACCI. The association between CCI and mortality was not statistically significant (*p* = 0.187). Detailed comparisons according to the CCI and ACCI categories are presented in Table 3 and Table 4 and Figure 2 and Figure 3, respectively.

Spearman’s rank correlation analyses showed nominal correlations of CCI with current age (ρ = 0.140, *p* = 0.013), age at IPF diagnosis (ρ = 0.152, *p* = 0.007), length of hospital stay (ρ = 0.169, *p* = 0.039), and baseline DLCO (ρ = −0.186, *p* = 0.026); however, none of these correlations remained statistically significant after Bonferroni correction (corrected significance threshold, *p* < 0.0036).

The ACCI was positively correlated with current age (ρ = 0.390, *p* < 0.001), age at IPF diagnosis (ρ = 0.420, *p* < 0.001), and the GAP index (ρ = 0.284, *p* = 0.002), all of which remained statistically significant after Bonferroni correction. A nominal inverse correlation was observed between the ACCI and baseline DLCO (ρ = −0.204, *p* = 0.014); however, this association was not statistically significant after correction. Neither the CCI nor the ACCI showed significant correlations with follow-up duration, annual acute exacerbation frequency, number of hospitalizations, overall survival, or the remaining baseline pulmonary function parameters evaluated (Table 5).

Kaplan–Meier survival analyses were performed to compare survival across the predefined CCI and ACCI score categories. No significant differences in survival were observed across the CCI categories (log-rank χ^2^ = 2.112, *p* = 0.549) or the ACCI categories (log-rank χ^2^ = 3.467, *p* = 0.325). These findings were consistent with the subsequent multivariable Cox proportional hazards regression analyses (Figure 4).

However, in multivariable Cox proportional hazards regression analyses, neither the CCI nor the ACCI was independently associated with survival. In the CCI model adjusted for age at diagnosis, baseline FVC, baseline DLCO, and antifibrotic treatment, the CCI was not an independent predictor of survival (HR = 1.029, 95% CI: 0.923–1.146; *p* = 0.610). Similarly, in the ACCI model adjusted for baseline FVC, baseline DLCO, and antifibrotic treatment, the ACCI was not independently associated with survival (HR = 1.035, 95% CI: 0.936–1.144; *p* = 0.509). Baseline DLCO was independently associated with survival in both models (CCI model: HR = 0.955, 95% CI, 0.935–0.976, *p* < 0.001; ACCI model: HR = 0.953, 95% CI, 0.933–0.974, *p* < 0.001) (Table 6).

### 3.3. Missing-Data and Sensitivity Analyses

Baseline DLCO was available for 143 of 319 patients (44.8%), whereas the GAP index was available for 114 patients (35.7%). Baseline FVC % predicted, FEV_1_% predicted, and FEV_1_/FVC were available for 210 (65.8%), 209 (65.5%), and 207 (64.9%) patients, respectively. Patients with and without available baseline DLCO data did not differ significantly in terms of age, age at IPF diagnosis, CCI, ACCI, acute exacerbation, hospitalization, LTOT requirement, or mortality. Similarly, no significant differences in these variables were observed between patients with and without GAP data. However, patients with available DLCO data had a longer median follow-up/survival time than those without DLCO data (46 [IQR, 27–75] vs. 30 [IQR, 18–54] months; *p* < 0.001) and more frequently received anti-fibrotic treatment (84.5% vs. 58.4%; *p* < 0.001). Similar differences were observed according to GAP availability, with longer median follow-up/survival among patients with available GAP data (49 [IQR, 29.3–77.3] vs. 30 [IQR, 19–57] months; *p* < 0.001) and a higher frequency of antifibrotic treatment (85.0% vs. 61.9%; *p* < 0.001) (Supplementary Table S1).

In sensitivity analyses excluding baseline DLCO from the Cox models, the complete-case sample increased from 133 to 206 patients (62 deaths). Neither CCI (HR = 1.025, 95% CI, 0.925–1.135; *p* = 0.641) nor ACCI (HR = 1.054, 95% CI, 0.962–1.155; *p* = 0.257) was independently associated with survival, consistent with the primary analyses.

## 4. Discussion

In this real-world cohort of 319 patients with IPF, comorbidities were common. After correction for multiple testing, higher CCI and ACCI scores remained significantly associated with incident malignancy and echocardiographic findings suggestive of pulmonary hypertension, whereas the previously observed associations with hospitalization did not retain statistical significance. In the correlation analyses, ACCI remained significantly correlated with current age, age at IPF diagnosis, and the GAP index after Bonferroni correction, whereas the nominal correlations of CCI and ACCI with baseline DLCO did not remain significant. Neither CCI nor ACCI was independently associated with survival in multivariable Cox regression analyses. Overall, these findings suggest that comorbidity indices may provide complementary information regarding clinical vulnerability in IPF but should be interpreted alongside disease-specific physiological measures rather than as stand-alone prognostic tools.

At least one comorbid condition was present in 75.5% of the cohort, with hypertension, coronary artery disease, and documented chronic obstructive pulmonary disease being the most frequent comorbidities. This profile is consistent with previous IPF cohorts, although the reported prevalence varies substantially according to the case definition and ascertainment methods. Kreuter et al. reported that 88% of patients with IPF had at least one comorbidity and demonstrated a graded reduction in median survival as the number of comorbidities increased [6]. Similarly, hypertension and chronic obstructive pulmonary disease were frequently reported in a nationwide Korean cohort [14]. Importantly, the observation that 27.0% of our patients were categorized as having no CCI-defined comorbidity despite a much higher prevalence of any recorded comorbidity is not contradictory. Several clinically relevant conditions in IPF, including hypertension, gastroesophageal reflux disease, obstructive sleep apnea, and pulmonary hypertension, are not represented in the original CCI. Fisher et al. similarly found a narrow CCI distribution in a large Canadian fibrotic ILD registry and cautioned that the index may incompletely represent the spectrum of comorbidities relevant to ILD [15]. Thus, the CCI should be understood as a weighted mortality-oriented index rather than a simple count of all coexisting diseases.

The marked shift from a median CCI of 2 to a median ACCI of 5, together with the increase in the proportion classified as severe from 18.2% using the CCI to 58.0% using the ACCI, was expected in this predominantly older population because age points are incorporated directly into the ACCI [9]. Consequently, a severe ACCI category should not be interpreted as indicating severe comorbidity alone; it represents the combined contribution of age and weighted comorbidities. This distinction is particularly important in IPF, in which advanced age is both a defining epidemiological feature and an established prognostic factor. In a cohort of older patients with ILD, Feltrer-Martinez et al. found that the age-modified Charlson score, but not the conventional Charlson score, was associated with mortality in the univariable analysis; however, only the composite physiologic index remained significant after multivariable adjustment [16]. This pattern is broadly concordant with our findings: although a nominal association between ACCI and mortality was observed before correction for multiple testing, this association did not remain statistically significant after Bonferroni correction, and neither ACCI nor CCI was independently associated with survival after multivariable adjustment. This further suggests that the apparent prognostic advantage of the ACCI may partly reflect its age-related component rather than an independent effect of comorbidity burden. Therefore, the observed correlations of the ACCI with current age, age at diagnosis, and GAP index are partly structural and should not be interpreted as independent biological relationships.

In the present study, a nominal association was observed between higher ACCI scores and mortality (unadjusted *p* = 0.044); however, this association did not remain statistically significant after Bonferroni correction. No significant association was observed between the conventional CCI and mortality. Published evidence regarding the prognostic role of the CCI in IPF and ILD has been inconsistent. Aoki et al. found no significant association between the CCI category and 3-year IPF-related events among 130 patients receiving antifibrotic therapy [10], which is consistent with our results for conventional CCI. Conversely, the cumulative comorbidity burden was associated with survival in the IPF cohort reported by Kreuter et al. [6]. Similarly, a recent real-world IPF cohort showed that higher cumulative comorbidity burden was independently associated with increased mortality [17]. These discrepancies may reflect differences in cohort composition, baseline physiological impairment, treatment selection, CCI thresholds, and outcome definitions of the studies.

Importantly, the apparent association between ACCI and mortality observed in the unadjusted analysis was not maintained after adjustment for relevant clinical and physiological variables. In the multivariable Cox regression analysis, neither CCI nor ACCI was independently associated with survival, whereas lower baseline DLCO remained independently associated with mortality in both models. Comparable findings were reported by Torres Tienza et al.; although survival differed significantly across Charlson categories in Kaplan–Meier analysis, the Charlson index was not independently associated with mortality in a multivariable Cox model adjusted for age, sex, FVC, and antifibrotic treatment [18].These findings suggest that the unadjusted association between ACCI and mortality may partly reflect the contribution of age and underlying physiological impairment rather than an independent prognostic effect of the comorbidity index itself. Accordingly, CCI and ACCI should be considered complementary measures of comorbidity burden rather than stand-alone predictors of survival in IPF.

Correlations with physiological measures provide further insights into the potential clinical role of comorbidity indices. Although nominal inverse correlations were observed between both CCI and ACCI and baseline DLCO, these associations did not remain statistically significant after Bonferroni correction. In contrast, the positive correlation between ACCI and the GAP index remained significant after correction. Neither index showed significant correlations with baseline FVC, FEV_1_, or FEV_1_/FVC. Previous studies have shown that incorporating CCI into disease-specific physiological models may improve prognostic performance in ILD [11,12]. Our findings support the rationale for such integrated approaches, although formal validation is required before their clinical application.

Hospitalization showed a nominal relationship with comorbidity burden, although the associations with CCI and ACCI did not remain statistically significant after Bonferroni correction. The hospitalization rate nevertheless increased across the CCI categories and reached 51.7% in the severe-CCI group; additionally, 66.4% of hospitalized patients were classified as having severe ACCI. These descriptive patterns should therefore be interpreted cautiously and require confirmation in larger cohorts. This finding is consistent with nationwide data from South Korea, in which a CCI of at least 4 was associated with greater healthcare utilization, including more hospitalizations and higher costs [14]. Spanish hospital discharge studies have likewise shown that hospitalized patients with IPF have longer stays and higher in-hospital mortality than matched patients without IPF, while also demonstrating that the CCI alone may not be the most discriminative comorbidity instrument for in-hospital mortality [19]. The association between CCI and ACCI and pulmonary hypertension in our cohort is also clinically relevant. Pulmonary hypertension is not a component of the original CCI, yet it is a recognized marker of advanced cardiopulmonary disease and was independently associated with in-hospital mortality among patients with IPF in a large Spanish analysis [20]. The clinical relevance of pulmonary hypertension is further supported by recent eurIPFreg data, in which PH was independently associated with reduced survival (HR 2.03) [21]. Therefore, its relationship with both indices may reflect the clustering of extrapulmonary disease, pulmonary vascular involvement, and more advanced overall illness in patients with IPF.

A particularly strong association was observed between severe CCI or ACCI categories and the development of malignancy during follow-up, and this association remained statistically significant after Bonferroni correction. This finding is clinically plausible because IPF and malignancy share several risk factors, including older age and smoking, and may involve common pathways related to abnormal tissue repair [6,10]. Kreuter et al. identified lung cancer as one of the comorbidities with the strongest adverse association with survival [6]. Nevertheless, this finding requires careful interpretation because solid tumors are weighted components of CCI. Therefore, the temporal distinction between malignancies present when the index was calculated and those arising subsequently is crucial. Although the present study calculated the CCI and ACCI using comorbidities documented at IPF diagnosis, retrospective uncertainty in the timing of cancer diagnosis could introduce incorporation or misclassification biases. Prospective studies using strictly time-stamped baseline comorbidities are needed to determine whether a higher baseline index truly identifies patients at an increased risk of subsequent malignancies.

Neither CCI nor ACCI differed according to the use or type of anti-fibrotic treatment. This finding suggests that within our center, the comorbidity burden did not clearly determine whether patients received pirfenidone or nintedanib. Trial data indicate that nintedanib consistently reduces FVC decline across subgroups defined by age, number of comorbidities, and CCI, although treatment discontinuation is more frequent among older patients and those with a greater comorbidity burden [22]. In contrast, analyses of large United States claims databases found that antifibrotic-treated patients were younger and had lower baseline CCI scores than untreated patients, indicating potential treatment-selection effects in routine practice [23]. Therefore, our findings should not be interpreted as demonstrating equal treatment tolerability or effectiveness across comorbidity strata because treatment allocation, dose modification, adherence, and discontinuation were not considered. Beyond currently established antifibrotic therapy, the therapeutic landscape of pulmonary fibrosis is evolving, with the recent approval of nerandomilast and ongoing phase III trials of additional agents [24].

Acute exacerbations have been reported in 2–16% of patients with IPF and are associated with high short-term mortality; the 16% frequency observed in our cohort was within this reported range [25]. Likewise, the absence of an association between either index and acute exacerbation is compatible with the lack of a CCI-related difference in 3-year IPF events reported by Aoki et al. [10]. Although Murohashi et al. found that CCI was prognostic in a heterogeneous population with acute or subacute interstitial pneumonias, CCI did not differ between survivors and non-survivors within the acute-exacerbation IPF subgroup [26]. The relatively small number of acute exacerbations in our cohort and reliance on retrospectively documented events may have also limited statistical power.

These findings may have several implications forclinical practice. The CCI offers a rapid estimate of the weighted comorbidity burden, whereas the ACCI incorporates the additional risk of age. They may help identify patients who warrant closer review of cardiovascular disease, pulmonary hypertension, malignancy risk, medication burden, and post-discharge needs. However, neither index captures the full comorbidity spectrum of IPF nor directly measures frailty, functional status, symptom burden, severity, or control of individual conditions. Frailty is also common in older patients with IPF; Sheth et al. reported frailty in 48% of their cohort, with frailty associated with lower lung function and a greater number of comorbidities [27]. Furthermore, because age is shared by the ACCI and GAP, using both indices without accounting for collinearity may overemphasize age. Therefore, a clinically useful approach would be to combine a comorbidity measure with disease-specific variables, such as the GAP stage and DLCO, rather than replacing the physiological assessment with a comorbidity index.

The strengths of this study include its relatively large real-world IPF cohort, direct head-to-head evaluation of the CCI and ACCI, and assessment of a broad range of functional and clinical outcomes of interest. This study had several limitations. First, the retrospective, single-center design is vulnerable to selection bias, incomplete documentation, and residual confounding, which may limit its generalizability. Second, substantial variable-specific missing data were present, particularly for baseline DLCO and the GAP index. Although patients with and without available DLCO or GAP data did not differ significantly in several major clinical characteristics and outcomes, including mortality, patients with available data had longer follow-up and were more frequently treated with antifibrotic therapy. Therefore, the missing data cannot be assumed to be completely random, and informative missingness and selection bias cannot be excluded. The precise reasons for individual missing measurements could not be systematically determined because of the retrospective study design. Nevertheless, sensitivity Cox analyses excluding baseline DLCO and thereby increasing the complete-case sample yielded results consistent with the primary analyses regarding the lack of an independent association of CCI or ACCI with survival. Third, comorbidities were determined from medical records without standardized prospective adjudication, and their severity, treatment, and temporal evolution were not considered in the analysis. Fourth, pulmonary hypertension was assessed using echocardiographic estimates of sPAP because right heart catheterization data were not available for all patients; therefore, some degree of misclassification cannot be excluded. Fifth, although time-to-event mortality was evaluated using multivariable Cox regression, the complete-case sample available for these models was reduced because of missing baseline pulmonary function data, particularly DLCO; therefore, the adjusted survival analyses should be interpreted cautiously. Cause-specific mortality data were also not available. In addition, the distribution of patients across the predefined ACCI categories was markedly unbalanced, particularly because the no-comorbidity category included only one patient and the mild category included relatively few patients. This limited the precision and statistical power of the category-based Kaplan–Meier and log-rank comparisons, and these findings should therefore be interpreted cautiously. Sixth, there was a markedly unbalanced distribution of ACCI categories, particularly the very small number of patients in the lower-score groups. Because age contributes directly to the ACCI, this distribution is expected in an older IPF population and may reduce the robustness of the categorical comparisons. Finally, treatment practices evolved during the long inclusion period, and confounding by indication may have influenced the comparisons involving anti-fibrotic therapy.

## 5. Conclusions

The comorbidity burden was prevalent and clinically relevant in this real-world IPF cohort. After correction for multiple testing, higher CCI and ACCI scores remained significantly associated with incident malignancy and echocardiographic findings suggestive of pulmonary hypertension, whereas the observed associations with hospitalization did not retain statistical significance. Although a nominal association was observed between ACCI and mortality, this association did not remain significant after Bonferroni correction, and neither CCI nor ACCI was independently associated with survival in multivariable Cox regression analyses. Lower baseline DLCO remained independently associated with survival in both Cox models. Overall, CCI and ACCI may complement disease-specific assessments based on pulmonary physiology but should not be regarded as stand-alone prognostic tools for IPF.

## Figures and Tables

**Figure 1:**
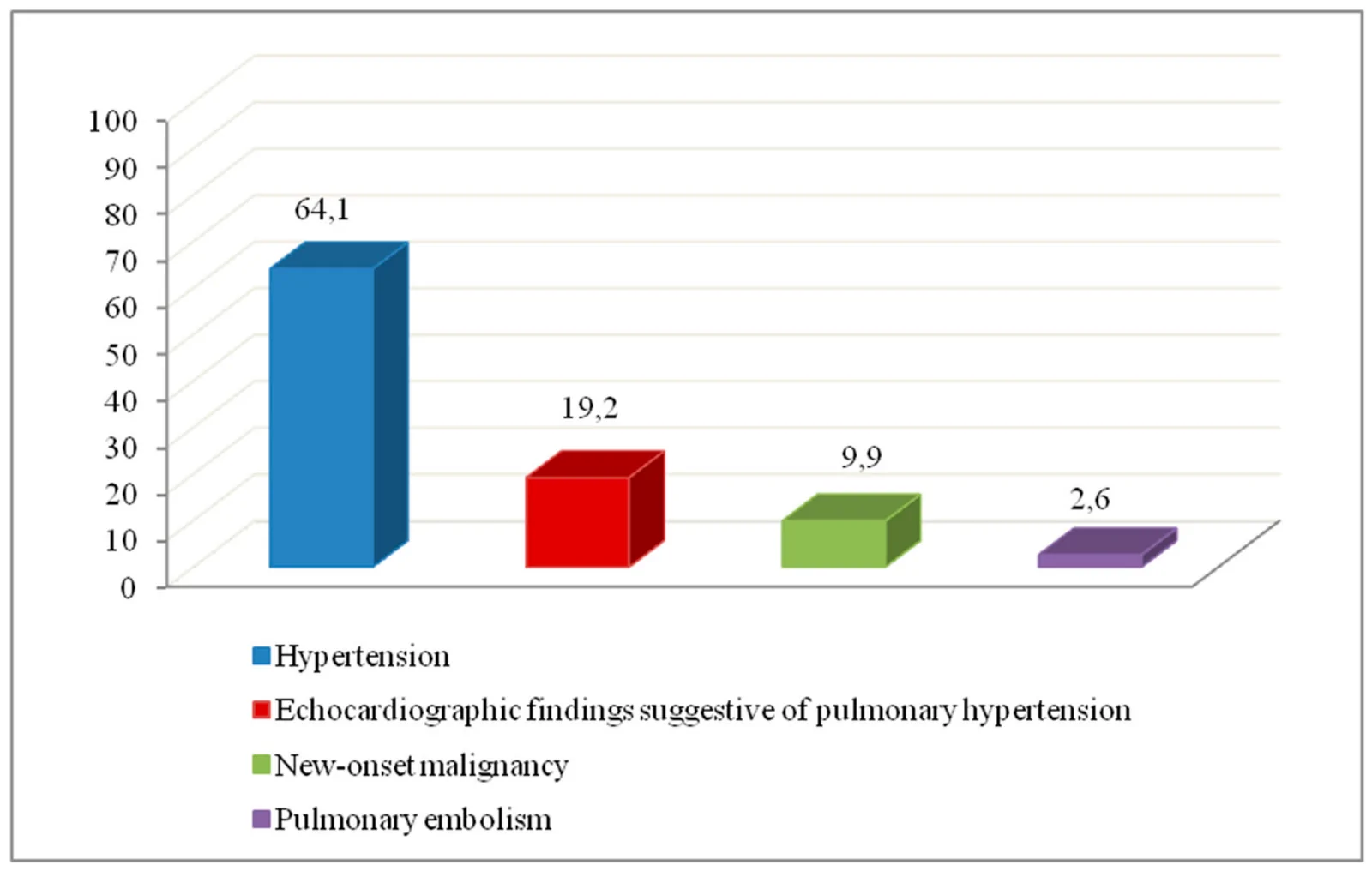
Prevalence of the Most Common Comorbidities Not Included in the CCI.

**Figure 2:**
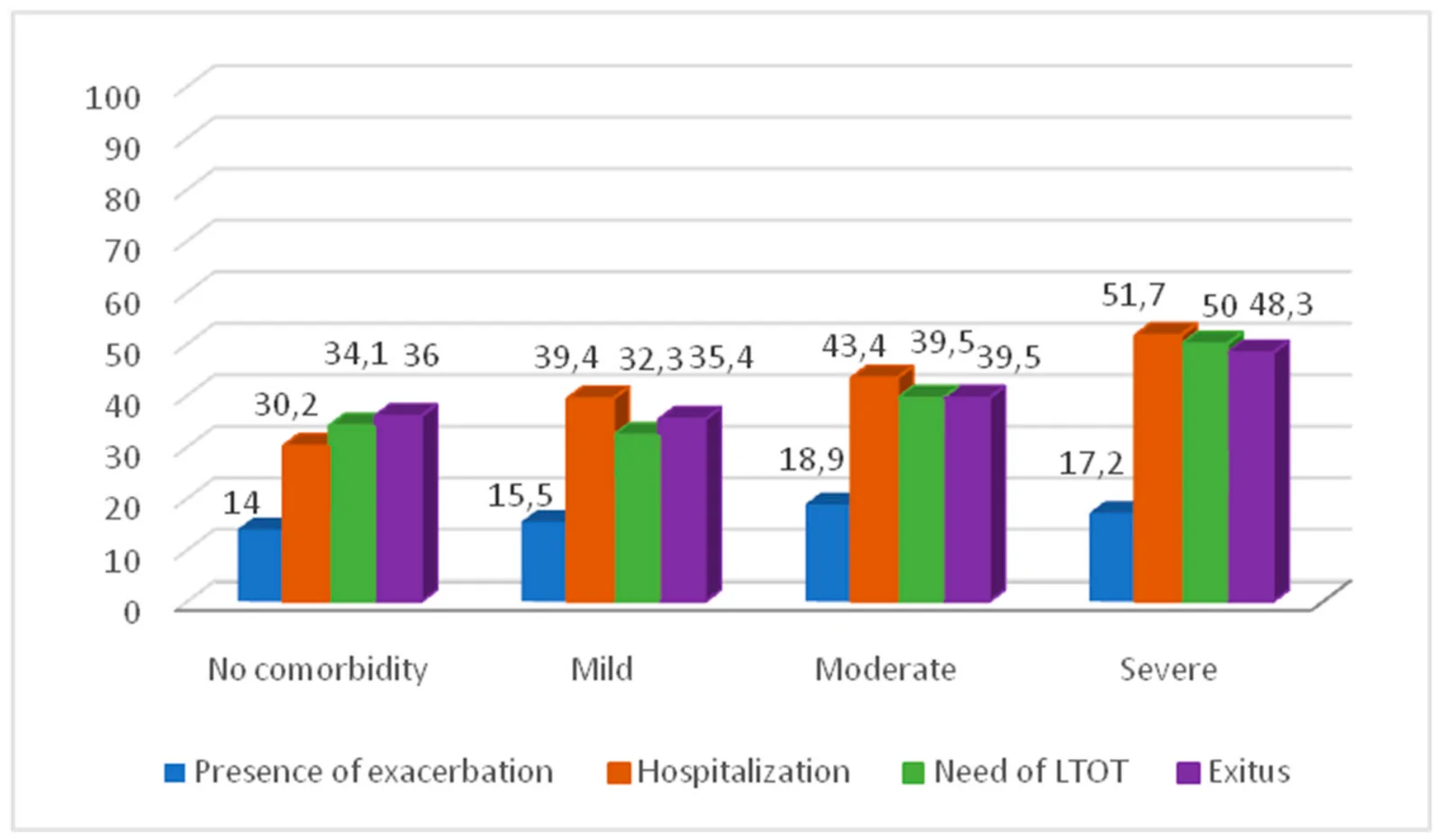
Clinical Outcomes and Mortality Rates According to the CCI Score Categories.

**Figure 3:**
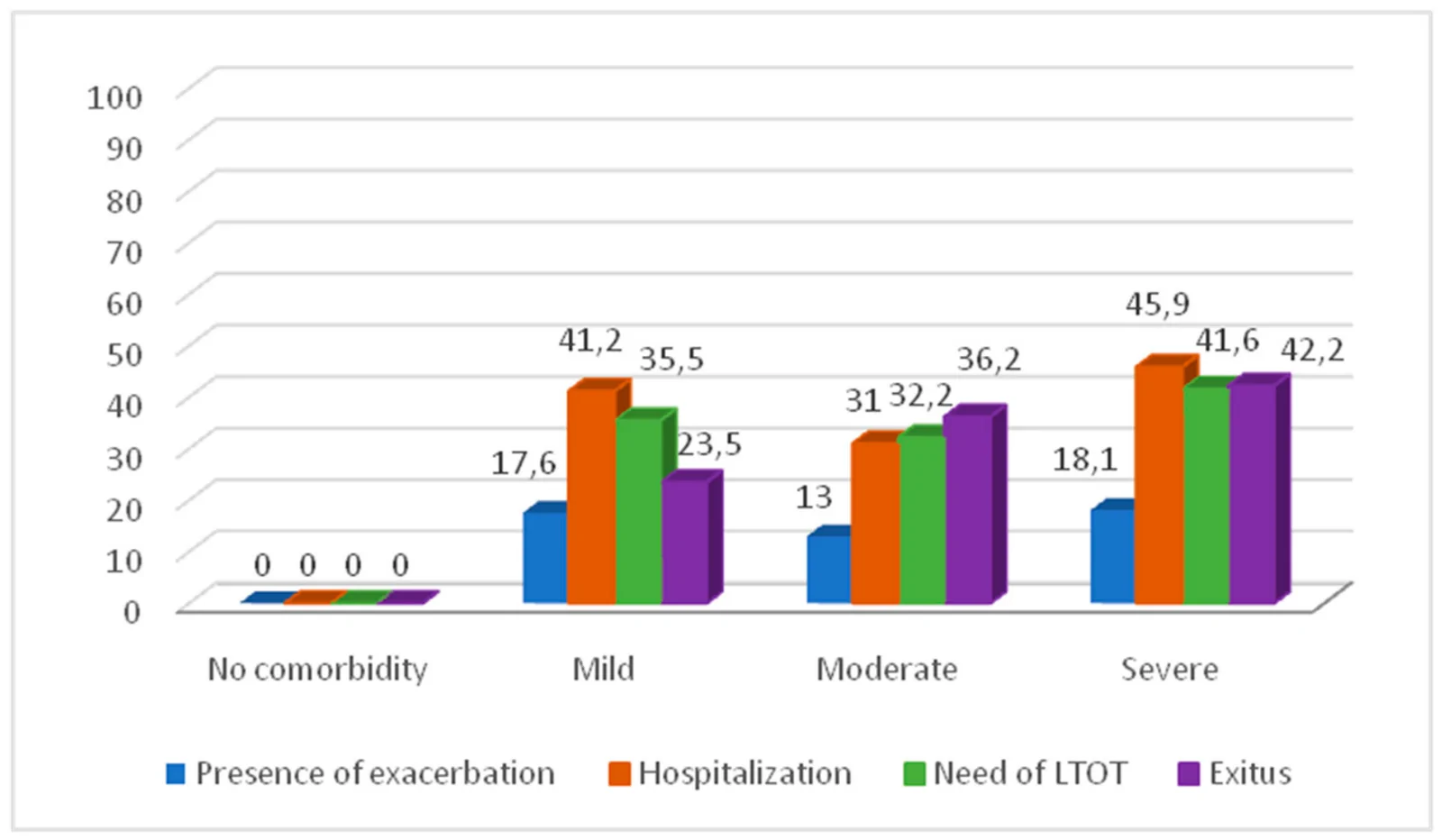
Clinical Outcomes and Mortality Rates According to the ACCI Score Categories.

**Figure 4:**
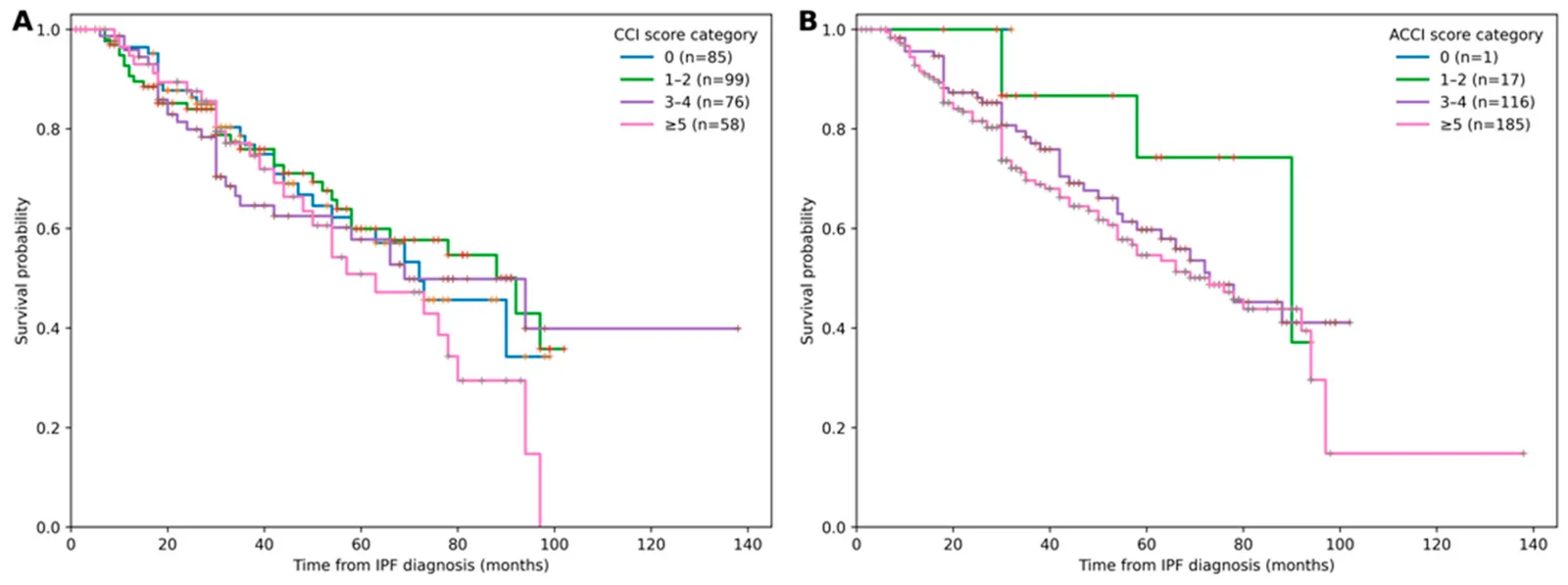
Kaplan–Meier survival curves according to comorbidity burden. (**A**) Survival according to Charlson Comorbidity Index (CCI) score categories (log-rank χ^2^ = 2.112, *p* = 0.549). (**B**) Survival according to Age-Adjusted Charlson Comorbidity Index (ACCI) score categories (log-rank χ^2^ = 3.467, *p* = 0.325). CCI and ACCI score categories were defined as 0, 1–2, 3–4, and ≥5. No statistically significant differences in survival were observed across the predefined categories for either index.

**Table 1:** Demographic Characteristics, CCI Scores, Pulmonary Function Parameters, and Clinical Outcomes of Patients with IPF.

*Demographic Characteristics*	*Category*		*n* (%)	
**Gender, *n* (%)**	Women		70 (22%)	
	Men		249 (78%)	
**Age, years, mean ± SD**			71 ± 8	
**Age of diagnosis, mean ± SD**			66 ± 5	
**<65 years, *n* (%)**			120 (37.6%)	
**≥65 years, *n* (%)**			199 (62.4%)	
**Presence of any comorbidity, *n* (%)**			241 (75.5%)	
**CCI score, Median/(25th–75thpercentiles)**			2 (0–4)	
**Age-adjusted CCI (ACCI) score, Median/(25th–75thpercentiles)**			5 (4–7)	
**CCI score classification**	No comorbidity		86 (27%)	
	Mild		99 (31%)	
	Moderate		76 (23.8%)	
	Severe		58 (18.2%)	
**Age-adjusted CCI (ACCI) classification**	No comorbidity		1 (0.3%)	
	Mild		17 (5.3%)	
	Moderate		116 (36.4%)	
	Severe		185 (58%)	
**GAP Index, mean ± SD**			3.6 ± 1.4	
***Pulmonary Function Parameters***				
	**Baseline**	**6th month**	**1st year**	**Last follow-up**
**FEV_1_ (ml), mean ± SD**	2175 ± 647	2296 ± 609	2306 ± 612	2105 ± 559
**FEV_1_ (%), mean ± SD**	80 ± 18	80 ± 16	82 ± 17	78 ± 19
**FVC (ml), mean ± SD**	2639 ± 824	2819 ± 792	2818 ± 812	2613 ± 763
**FVC (%), mean ± SD**	76 ± 18	78 ± 17	80 ± 17	75 ± 18
**FEV_1_/FVC (%), mean ± SD**	84 ± 11	82 ± 10	82 ± 8	82 ± 8
**DLCO (%), mean ± SD**	51 ± 16	49 ± 16	54 ± 12	45 ±13
***Treatment***				
**Pharmacological treatment, *n* (%)**	Yes		221 (70.2%)	
	No		94 (29.8%)	
**Antifibrotic medication, *n* (%)**	Pirfenidone		125 (56.6%)	
	Nintedanib		96 (43.4)	
***Clinical outcomes***				
**Presence of exacerbation, *n* (%)**			51 (16%)	
**Hospitalization, *n* (%)**			128 (40.1%)	
**Total days of hospitalization, days, mean ± SD**			14 ± 20	
**Need of LTOT, *n* (%)**			120 (37.7%)	
**Overall survival, months**			43.8 (±26.6)	
**Exitus, *n* (%)**			124 (38.9%)	

**Table 2:** Distribution of Comorbidities Included in the CCI Among Patients with IPF.

	*n*	%
**Myocardial infarction**	135	43.4
**Congestive Heart Failure**	46	14.8
**Peripheral VascularDisease**	13	4.2
**Cerebrovascular Disease**	28	9
**Dementia**	10	3.2
**COPD**	94	30.3
**Rheumatic disease**	11	3.5
**Peptic Ulcer**	20	6.4
**Chronic liver disease**	12	3.9
**Diabetes mellitus**	96	30.6
**Hemiplegia**	3	1
**Chronic renal disease**	46	14.8
**Solid organ tumor**	53	17
**Leukemia/Lymphoma**	5	1.5
**AIDS**	0	0

**Table 3:** Distribution of demographic characteristics, clinical findings, and outcomes of patients with IPF stratified by CCI score categories.

	No Comorbidity (*n*:86)	Mild (*n*:99)	Moderate (*n*:76)	Severe (*n*:58)	*p*
**Women, *n* (%)**	21(24.4%)	27 (27.3%)	15 (19.7%)	7 (12.1%)	0.141
**Men, *n* (%)**	65 (75.6%)	72 (72.7%)	61 (80.3%)	51 (87.9%)	
**Age of diagnosis (years)**					
**<65 years**	45 (52.3%)	34 (34.3%)	26 (34.2%)	15 (25.9%)	**<0.001**
**≥65 years**	41 (47.7%)	65 (65.7%)	50 (65.8%)	43 (74.1%)	
**Antifibroticmedication, *n* (%)**					
**Yes**	61 (73.5%)	69 (70.4%)	54 (71.1%)	37 (63.8%)	0.66
**No**	22 (26.5%)	29 (29.6%)	22 (28.9%)	21 (36.2%)	
**Antifibrotic medication, *n* (%)**					
**Pirfenidone**	30 (49.2%)	40 (58%)	36 (66.7%)	19 (51.4%)	0.255
**Nintedanib**	31 (50.8%)	29 (42%)	18 (33.3%)	18 (48.6%)	
**Presence of exacerbation, *n* (%)**					
**Yes**	12 (14%)	15 (15.5%)	14 (18.9%)	10 (17.2%)	0.591
**No**	74 (86%)	82 (84.5%)	60 (81.1%)	48 (82.8%)	
**Hospitalization, *n* (%)**					
**Yes**	26 (30.2%)	39 (39.4%)	33 (43.4%)	30 (51.7%)	**0.006**
**No**	60 (69.8%)	60 (60.6%)	43 (56.6%)	28 (48.3%)	
**New-onset lung malignancy, *n* (%)**					
**Yes**	1 (1.2%)	6 (6.1%)	1 (1.4%)	23 (41.1%)	**<0.001**
**No**	85 (98.8%)	93 (93.9%)	72 (98.6%)	33 (58.9%)	
**Echocardiographic findings suggestive ofpulmonary hypertension, *n* (%)**					
**Yes**	6 (7.5%)	19 (19.4%)	21 (27.6%)	14 (24.1%)	**0.002**
**No**	74 (92.5%)	79 (80.6%)	55 (72.4%)	44 (75.9%)	
**Need of LTOT, *n* (%)**					
**Yes**	29 (34.1%)	32 (32.3%)	30 (39.5%)	29 (50.0%)	0.138
**No**	56 (65.9%)	67 (67.7%)	46 (60.5%)	29 (50.0%)	
**Exitus, *n* (%)**					
**Yes**	31 (36.0%)	35 (35.4%)	30 (39.5%)	28 (48.3%)	
**No**	55 (64.0%)	64 (64.6%)	46 (60.5%)	30 (51.7%)	0.187

Bonferroni-corrected significance threshold: *p* < 0.005 (0.05/10 comparisons).

**Table 4:** Distribution of demographic characteristics, clinical findings, and outcomes of patients with IPF stratified by ACCI score categories.

	No Comorbidity (*n*:1)	Mild (*n*:17)	Moderate (*n*:116)	Severe (*n*:185)	*p*
**Women, *n* (%)**	0(0%)	2 (11.8%)	32 (27.6%)	36 (19.5%)	0.248
**Men, *n* (%)**	1 (100%)	15 (88.2%)	84 (72.4%)	149 (80.5%)	
**Age of diagnosis (years)**					
**<65 years**	1(100%)	16 (94.1%)	52 (44.8%)	51 (27.6%)	**<0.001**
**≥65 years**	0 (0%)	1 (5.9%)	64 (55.2%)	134 (72.4%)	
**Pharmacological treatment, *n* (%)**					
**Yes**	1(100%)	11 (68.8%)	86 (76.1%)	123 (66.5%)	0.315
**No**	0 (0%)	6 (31.2%)	27 (23.9%)	62 (33.5%)	
**Antifibrotic medication, *n* (%)**					
**Pirfenidone**	0(0%)	6 (54.5%)	46 (53.5%)	73 (59.5%)	0.564
**Nintedanib**	1 (100%)	5(45.5%)	40 (46.5%)	50 (40.7%)	
**Presence of exacerbation, *n* (%)**					
**Yes**	0(0%)	3 (17.6%)	15 (13.0%)	33 (18.1%)	0.467
**No**	1 (100%)	14 (82.4%)	100 (87.0%)	149 (81.9%)	
**Hospitalization, *n* (%)**					
**Yes**	0(0%)	7 (41.2%)	36 (31.0%)	85 (45.9%)	**0.011**
**No**	1 (100%)	10 (58.8%)	80 (69.0%)	100 (54.1%)	
**New-onset lung malignancy, *n* (%)**					
**Yes**	0(0%)	0 (0%)	4 (3.4%)	27 (15.0%)	**<0.001**
**No**	1 (100%)	17 (100%)	112 (96.6%)	153 (85.0%)	
**Echocardiographic findings suggestive ofpulmonary hypertension, *n* (%)**					
**Yes**	0(0%)	0 (0%)	15 (13.5%)	45 (24.5%)	**0.002**
**No**	1 (100%)	17 (100%)	96 (86.5%)	139 (75.5%)	
**Need of LTOT, *n* (%)**					
**Yes**	0(0%)	6 (35.5%)	37 (32.2%)	77 (41.6%)	0.340
**No**	1 (100%)	11 (64.7%)	78 (67.8%)	108 (58.4%)	
**Exitus, *n* (%)**					
**Yes**	0(0%)	4 (23.5%)	42 (36.2%)	78 (42.2%)	0.044
**No**	1 (100%)	13 (76.5%)	74 (63.8%)	107 (57.8%)	

Bonferroni-corrected significance threshold: *p* < 0.005 (0.05/10 comparisons).

**Table 5:** Spearman rank correlation analysis between CCI and ACCI scores and demographic, clinical, and pulmonary function parameters in patients with IPF.

Parameters	CCI Score			Age-Adjusted CCI Score (ACCI)		
	Spearman Rank Correlation	*p*	*n*	Spearman Rank Correlation	*p*	*n*
**Age**	0.140	**0.013**	319	0.390	**<0.001**	319
**Age of diagnosis**	0.152	**0.007**	319	0.420	**<0.001**	319
**Mean follow-up duration (months)**	−0.003	0.954	318	−0.054	0.336	318
**Number of acute exacerbations per year**	−0.023	0.824	97	−0.011	0.915	97
**GAP Index**	0.179	0.056	114	0.284	**0.002**	114
**Number of hospitalizations**	0.105	0.196	155	0.066	0.414	155
**Length of hospital stay, (days)**	0.169	**0.039**	152	0.124	0.127	152
**Overall survival, (months)**	0.004	0.947	319	−0.052	0.353	319
**Baseline FEV_1_ (mL)**	−0.016	0.824	188	−0.092	0.212	188
**Baseline FEV_1_ (% predicted**	−0.080	0.252	209	−0.079	0.255	209
**Baseline FVC (mL)**	−0.003	0.972	188	−0.067	0.361	188
**Baseline FVC (% predicted)**	−0.059	0.395	210	−0.081	0.242	210
**Baseline FEV_1_/FVC (%)**	−0.119	0.089	207	−0.126	0.070	207
**Baseline DLCO (% predicted)**	−0.186	**0.026**	143	−0.204	**0.014**	143

Bonferroni-corrected significance threshold: *p* < 0.0036 (0.05/14 correlations for each index).

**Table 6:** Multivariable Cox proportional hazards regression analyses for survival.

Model/Variable	HR	95% CI	*p*-Value
**Model 1: CCI-based model (*n* = 133; deaths = 47)**			
CCI	1.029	0.923–1.146	0.610
Age at diagnosis, years	1.039	0.986–1.096	0.153
Baseline FVC, % predicted	0.990	0.972–1.010	0.328
Baseline DLCO, % predicted	**0.955**	**0.935–0.976**	**<0.001**
Antifibrotic treatment	0.898	0.380–2.123	0.806
**Model 2: ACCI-based model (*n* = 133; deaths = 47)**			
ACCI	1.035	0.936–1.144	0.509
Baseline FVC, % predicted	0.988	0.969–1.007	0.215
Baseline DLCO, % predicted	**0.953**	**0.933–0.974**	**<0.001**
Antifibrotic treatment	0.781	0.338–1.803	0.562

## Data Availability

The data presented in this study are available from the corresponding author upon reasonable request. The data are not publicly available because of patient privacy and institutional/ethical restrictions.

## Supplementary Materials

The following supporting information can be downloaded at https://www.mdpi.com/article/10.3390/biomedicines14102176/s1, Table S1. Missing-data assessment and comparison of patients according to the availability of baseline DLCO and GAP data.

## Abbreviations

ACCI	Age-Adjusted Charlson Comorbidity Index
AIDS	Acquired immunodeficiency syndrome
CCI	Charlson Comorbidity Index
COPD	Chronic Obstructive Pulmonary Disease
CI	Confidence interval
DLCO	Diffusing capacity of the lung for carbon monoxide
FEV_1_	Forced expiratory volume in one second
FVC	Forced vital capacity
GAP	Gender–Age–Physiology
HR	Hazard ratio
HRCT	High-resolution computed tomography
ILD	Interstitial lung disease
IPF	Idiopathic pulmonary fibrosis
IQR	Interquartile range
LTOT	Long-term oxygen therapy
SD	Standard deviation
sPAP	Systolic pulmonary artery pressure
UIP	Usual interstitial pneumonia

## References

[1] Raghu G., Remy-Jardin M., Myers J.L., Richeldi L., Ryerson C.J., Lederer D.J., Behr J., Cottin V., Danoff S.K., Morell F. (2018). Diagnosis of Idiopathic Pulmonary Fibrosis. An Official ATS/ERS/JRS/ALAT Clinical Practice Guideline. Am. J. Respir. Crit. Care Med..

[2] Raghu G., Remy-Jardin M., Richeldi L., Thomson C.C., Inoue Y., Johkoh T., Kreuter M., Lynch D.A., Maher T.M., Martinez F.J. (2022). Idiopathic Pulmonary Fibrosis (an Update) and Progressive Pulmonary Fibrosis in Adults: An Official ATS/ERS/JRS/ALAT Clinical Practice Guideline. Am. J. Respir. Crit. Care Med..

[3] King T.E., Bradford W.Z., Castro-Bernardini S., Fagan E.A., Glaspole I., Glassberg M.K., Gorina E., Hopkins P.M., Kardatzke D., Lancaster L. (2014). A Phase 3 Trial of Pirfenidone in Patients with Idiopathic Pulmonary Fibrosis. N. Engl. J. Med..

[4] Richeldi L., Du Bois R.M., Raghu G., Azuma A., Brown K.K., Costabel U., Cottin V., Flaherty K.R., Hansell D.M., Inoue Y. (2015). Correction: Efficacy and Safety of Nintedanib in Idiopathic Pulmonary Fibrosis. N. Engl. J. Med..

[5] Raghu G., Amatto V.C., Behr J., Stowasser S. (2015). Comorbidities in Idiopathic Pulmonary Fibrosis Patients: A Systematic Literature Review. Eur. Respir. J..

[6] Kreuter M., Ehlers-Tenenbaum S., Palmowski K., Bruhwyler J., Oltmanns U., Muley T., Heussel C.P., Warth A., Kolb M., Herth F.J.F. (2016). Impact of Comorbidities on Mortality in Patients with Idiopathic Pulmonary Fibrosis. PLoS ONE.

[7] Ley B., Ryerson C.J., Vittinghoff E., Ryu J.H., Tomassetti S., Lee J.S., Poletti V., Buccioli M., Elicker B.M., Jones K.D. (2012). A Multidimensional Index and Staging System for Idiopathic Pulmonary Fibrosis. Ann. Intern. Med..

[8] Charlson M.E., Pompei P., Ales K.L., MacKenzie C.R. (1987). A New Method of Classifying Prognostic Comorbidity in Longitudinal Studies: Development and Validation. J. Chronic Dis..

[9] Charlson M., Szatrowski T.P., Peterson J., Gold J. (1994). Validation of a Combined Comorbidity Index. J. Clin. Epidemiol..

[10] Aoki A., Hara Y., Fujii H., Murohashi K., Nagasawa R., Tagami Y., Enomoto T., Matsumoto Y., Masuda M., Watanabe K. (2023). The Clinical Impact of Comorbidities among Patients with Idiopathic Pulmonary Fibrosis Undergoing Anti-Fibrotic Treatment: A Multicenter Retrospective Observational Study. PLoS ONE.

[11] Yagyu H., Murohashi K., Hara Y., Saigusa Y., Aoki A., Kobayashi N., Kaneko T. (2020). Clinical Utility of a Composite Scoring System Including Charlson Comorbidity Index Score in Patients with Interstitial Lung Disease. J. Thorac. Dis..

[12] Fujii H., Hara Y., Saigusa Y., Tagami Y., Murohashi K., Nagasawa R., Aoki A., Izawa A., Seki K., Watanabe K. (2023). ILD-GAP Combined with the Charlson Comorbidity Index Score (ILD-GAPC) as a Prognostic Prediction Model in Patients with Interstitial Lung Disease. Can. Respir. J..

[13] Song J.W., Song J.-K., Kim D.S. (2009). Echocardiography and Brain Natriuretic Peptide as Prognostic Indicators in Idiopathic Pulmonary Fibrosis. Respir. Med..

[14] Lee J.H., Park H.J., Kim S., Kim Y.-J., Kim H.C. (2023). Epidemiology and Comorbidities in Idiopathic Pulmonary Fibrosis: A Nationwide Cohort Study. BMC Pulm. Med..

[15] Fisher J.H., Kolb M., Algamdi M., Morisset J., Johannson K.A., Shapera S., Wilcox P., To T., Sadatsafavi M., Manganas H. (2019). Baseline Characteristics and Comorbidities in the CAnadianREgistry for Pulmonary Fibrosis. BMC Pulm. Med..

[16] Feltrer-Martínez L., Orozco S., Alonso A., Millan-Billi P., Barril S., Ruibal G., Francesqui J., Lobo-Prat D., Gimenez A., Lopez L. (2024). Older Patients with Interstitial Lung Disease Feature a Distinct Clinical Profile. Open Respir. Arch..

[17] Shen Q., Zhou S., Ouyang X., Tan Y., Chen S., Shi J., Peng H. (2026). Comorbidity Burden and Survival in Patients with Idiopathic Pulmonary Fibrosis: A Real-World Cohort Study. Heart Lung.

[18] Torres Tienza S., de Miguel-Díez J., Ortega C.G., Jareño Esteban J.J. (2026). Comorbidity in Patients with Idiopathic Pulmonary Fibrosis: Evaluation Using the Charlson, TORVAN and GAP Indices. J. Clin. Med..

[19] Pedraza-Serrano F., Jiménez-García R., López-de-Andrés A., Hernández-Barrera V., Esteban-Hernández J., Sánchez-Muñoz G., Puente-Maestu L., De-Miguel-Díez J. (2018). Comorbidities and Risk of Mortality among Hospitalized Patients with Idiopathic Pulmonary Fibrosis in Spain from 2002 to 2014. Respir. Med..

[20] Pedraza-Serrano F., Jiménez-García R., López-de-Andrés A., Hernández-Barrera V., Sánchez-Muñoz G., Puente-Maestu L., De-Miguel-Díez J. (2019). Characteristics and Outcomes of Patients Hospitalized with Interstitial Lung Diseases in Spain, 2014 to 2015. Medicine.

[21] Guenther A., Tello S., Schoppe M.C., Pons-Kuehnemann J., Seeger W., Stiben J., Tello K., Molina Molina M., Vancheri C., Crestani B. (2025). Pulmonary Hypertension Drives Prognosis in Idiopathic Pulmonary Fibrosis: Insights from the European IPF Registry. J. Clin. Med..

[22] Glaspole I., Bonella F., Bargagli E., Glassberg M.K., Caro F., Stansen W., Quaresma M., Orsatti L., Bendstrup E. (2021). Efficacy and Safety of Nintedanib in Patients with Idiopathic Pulmonary Fibrosis Who Are Elderly or Have Comorbidities. Respir. Res..

[23] Qiu Y., Zhu J., Chopra P., Elpers B., Dieyi C., Byrne C., Tang J., Wang Y., Govindaraj K., Fischer A. (2024). Real-World Antifibrotic Treatment Patterns in Patients with Idiopathic Pulmonary Fibrosis: Retrospective Analyses of Two Large Healthcare Administrative Databases in the United States. Ther. Adv. Respir. Dis..

[24] Carriera L., Lipsi R., Dodaj M., Inchingolo R., Smargiassi A., Coppola A., Mari P.-V., Barone R., Ielo S., Scala R. (2026). Most Promising Emerging Therapies for Pulmonary Fibrosis: Targeting Novel Pathways. Biomedicines.

[25] Mari P.-V., Coppola A., Carriera L., Macagno F. (2025). The Impact of Corticosteroids on Mortality in Acute Exacerbations of Idiopathic Pulmonary Fibrosis: A Meta-Analysis. Adv. Respir. Med..

[26] Murohashi K., Hara Y., Saigusa Y., Kobayashi N., Sato T., Yamamoto M., Kudo M., Kaneko T. (2019). Clinical Significance of Charlson Comorbidity Index as a Prognostic Parameter for Patients with Acute or Subacute Idiopathic Interstitial Pneumonias and Acute Exacerbation of Collagen Vascular Diseases-Related Interstitial Pneumonia. J. Thorac. Dis..

[27] Sheth J.S., Xia M., Murray S., Martinez C.H., Meldrum C.A., Belloli E.A., Salisbury M.L., White E.S., Holtze C.H., Flaherty K.R. (2019). Frailty and Geriatric Conditions in Older Patients with Idiopathic Pulmonary Fibrosis. Respir. Med..

