# Supplementary material for: Association of the Charlson Comorbidity Index and Age-Adjusted Charlson Comorbidity Index with Clinical Course and Mortality in Patients with Idiopathic Pulmonary Fibrosis
Source: Biomedicines. 2026 Sep 25;14(10):2176. doi: 10.3390/biomedicines14102176 (PMC13701863; doi:10.3390/biomedicines14102176)
Supplement: Supplementary file 1 [file biomedicines-14-02176-s001.zip › biomedicines-4558763-supplementary.pdf]

Supplementary Table S1. Missing-data assessment and comparison of patients according to the availability of baseline DLCO and GAP data

A. Availability of key disease-specific variables

Variable	Available, n (%)	Missing, n (%)
Baseline DLCO, % predicted	143 (44.8)	176 (55.2)
GAP index	114 (35.7)	205 (64.3)
Baseline FVC, % predicted	210 (65.8)	109 (34.2)
Baseline FEV_1, % predicted	209 (65.5)	110 (34.5)
Baseline FEV_1/FVC	207 (64.9)	112 (35.1)

B. Comparison according to baseline DLCO availability

Variable	DLCO available (n=143)	DLCO missing (n=176)	p-value
Current age, years	71 (68–76)	72 (66.8–78)	0.472
Age at IPF diagnosis, years	67 (62.5–71)	68.5 (63–73)	0.093
CCI	2 (0–4)	2 (0–4)†	0.299
ACCI	5 (4–7)	5 (4–7)	0.356
Antifibrotic treatment	120/142 (84.5%)	101/173 (58.4%)	**<0.001**
Acute exacerbation	24/142 (16.9%)	27/173 (15.6%)	0.876
Hospitalization	59/143 (41.3%)	69/176 (39.2%)	0.797
LTOT requirement	56/142 (39.4%)	64/176 (36.4%)	0.656
Mortality	53/143 (37.1%)	71/176 (40.3%)	0.630
Follow-up/survival time, months	46 (27–75)	30 (18–54)	**<0.001**

† CCI was available for 175 of the 176 patients with missing DLCO.

C. Comparison according to GAP availability

Variable	GAP available (n=114)	GAP missing (n=205)	p-value
Current age, years	71.5 (67–75.8)	72 (67–78)	0.464
Age at IPF diagnosis, years	67 (62–71)	68 (64–73)	0.064
CCI	2 (0.25–4)	2 (0–4)†	0.289
ACCI	5 (4–7)	5 (4–7)	0.433
Antifibrotic treatment	96/113 (85.0%)	125/202 (61.9%)	**<0.001**
Acute exacerbation	17/113 (15.0%)	34/202 (16.8%)	0.800
Hospitalization	45/114 (39.5%)	83/205 (40.5%)	0.954
LTOT requirement	46/113 (40.7%)	74/205 (36.1%)	0.490
Mortality	44/114 (38.6%)	80/205 (39.0%)	1.000
Follow-up/survival time, months	49 (29.3–77.3)	30 (19–57)	**<0.001**

† CCI was available for 204 of the 205 patients with missing GAP data

Data are presented as median (interquartile range) or n/N (%), as appropriate. Continuous variables were compared using the Mann–Whitney U test, and categorical variables were compared using the chi-square test. Denominators vary because of variable-specific missing data.
